# p110δ PI3 kinase pathway: emerging roles in cancer

**DOI:** 10.3389/fonc.2013.00040

**Published:** 2013-03-01

**Authors:** Niki Tzenaki, Evangelia A. Papakonstanti

**Affiliations:** Department of Biochemistry, School of Medicine, University of CreteHeraklion, Greece

**Keywords:** p110δ PI3K, cancer, hematologic malignancies, PTEN, solid tumors

## Abstract

Class IA PI3Ks consists of three isoforms of the p110 catalytic subunit designated p110α, p110β, and p110δ which are encoded by three separate genes. Gain-of-function mutations on *PIK3CA* gene encoding for p110α isoform have been detected in a wide variety of human cancers whereas no somatic mutations of genes encoding for p110β or p110δ have been reported. Unlike p110α and p110β which are ubiquitously expressed, p110δ is highly enriched in leukocytes and thus the p110δ PI3K pathway has attracted more attention for its involvement in immune disorders. However, findings have been accumulated showing that the p110δ PI3K plays a seminal role in the development and progression of some hematologic malignancies. A wealth of knowledge has come from studies showing the central role of p110δ PI3K in B-cell functions and B-cell malignancies. Further data have documented that wild-type p110δ becomes oncogenic when overexpressed in cell culture models and that p110δ is the predominant isoform expressed in some human solid tumor cells playing a prominent role in these cells. Genetic inactivation of p110δ in mice models and highly-selective inhibitors of p110δ have demonstrated an important role of this isoform in differentiation, growth, survival, motility, and morphology with the inositol phosphatase PTEN to play a critical role in p110δ signaling. In this review, we summarize our understanding of the p110δ PI3K signaling pathway in hematopoietic cells and malignancies, we highlight the evidence showing the oncogenic potential of p110δ in cells of non-hematopoietic origin and we discuss perspectives for potential novel roles of p110δ PI3K in cancer.

## General aspects of the class IA PI3Ks signaling pathway

**Figure 1:**
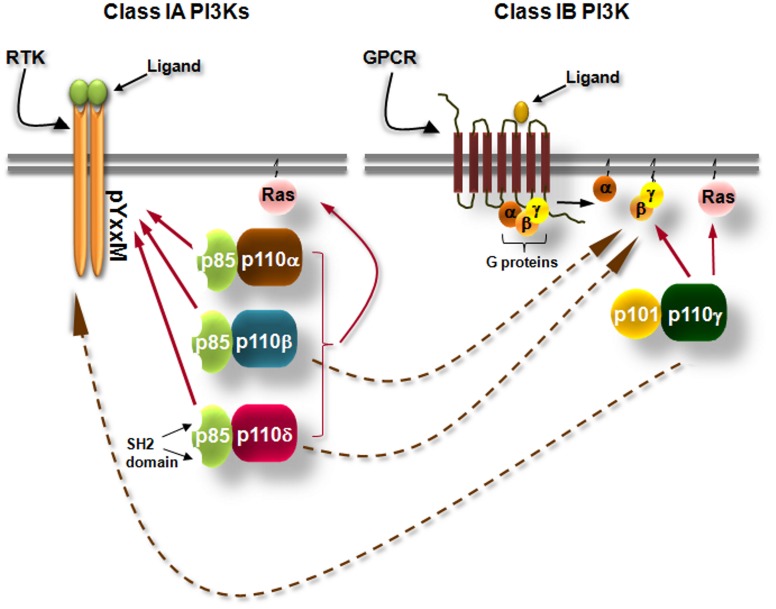
**Simplified scheme showing the differential activation of class IA and class IB PI3K isoforms.** Class IA PI3Ks are heterodimers consisting of a 110 kDa catalytic subunit (p110α, p110β, and p110δ) in complex with a p85 regulatory subunit, of which five isoforms exist. Class IA PI3Ks are activated by growth factor and cytokine receptors or adaptor proteins (e.g., CD19/BCAP in B cells). Binding of the ligand to its receptor leads to receptor dimerization and auto-phosphorylation of tyrosines (Y) which are located in pYxxM motifs. The p85 regulatory subunits have Src-homology 2 (SH2) domains which bind to phosphorylated tyrosines in YxxM motifs recruiting thus the class IA PI3Ks to the plasma membrane where their lipid substrates are located. Class IB PI3K consists of the p110γ isoform which binds to p101 or p84 regulatory subunits. Class IB PI3K is activated by G protein-coupled receptors (GPCRs). Binding of the ligand (e.g., a chemokine) to its cognate GPCR induces the dissociation of heterotrimeric G-proteins and the Gβγ subunits interact with the class IB PI3K. Arrows with dashed lines represent activation of p110β and p110δ downstream of GPCRs and activation of p110γ downstream of tyrosine kinases by currently unknown mechanisms.

Class I phosphoinositide-3 kinases (PI3Ks) consist of a group of enzymes that transmit signals inside cells by the production of intracellular second messenger lipid signals. PI3Ks phosphorylate inositol lipids at the 3-position of the inositol ring, generating phosphatidylinositol (PI)-3-phosphate (PI3P), phosphatidylinositol-3,4-bisphosphate [PI(3,4)P_2_] and phosphatidyl-inositol-3,4,5-trisphosphate [PI(3,4,5)P_3_]. These lipids trigger signal transduction cascades that control cell division, survival, metabolism, intracellular trafficking, differentiation, re-organization of the actin cytoskeleton, and cell migration under the control of PI3Ks (Vanhaesebroeck et al., 2001; Hawkins et al., 2006; Low et al., 2010; Zwaenepoel et al., 2012). The PI3K isoforms that are activated by tyrosine kinases and G-protein coupled receptors (GPCRs) are known as class IA and IB PI3Ks, respectively (Figure 1). Class IA PI3Ks are constitutive heterodimers of a 110 kDa catalytic subunit (p110) with one of the five regulatory adaptor proteins (p85α, p55α, p50α, p85β, or p55γ, collectively called “p85s”) that recruits the p110 to intracellular locations of tyrosine kinase activation (Vanhaesebroeck et al., 1997a, 2010) (Figure 1). Mammals have genes for 3 class IA catalytic subunits designated p110α, p110β, and p110δ (Vanhaesebroeck et al., 2010) (Figure 1). p110γ is the only class IB PI3K. This kinase occurs in complex with the p101 (Stephens et al., 1997; Krugmann et al., 1999) or p84 (Suire et al., 2005; Voigt et al., 2006) adaptor protein and is activated by the Gβγ subunits of heterotrimeric G-proteins (Figure 1). However, several studies have linked the p110β and p110δ isoforms of class IA to GPCRs and the class IB p110γ to tyrosine kinases, the mechanisms though are not yet clear (Sadhu et al., 2003; Reif et al., 2004; Condliffe et al., 2005; Guillermet-Guibert et al., 2008; Durand et al., 2009; Hoellenriegel et al., 2011; Schmid et al., 2011) (Figure 1). All catalytic subunits of the class I PI3Ks contain binding domains for Ras GTPases (Figure 1) and their binding to certain Ras proteins contributes to activation (Rodriguez-Viciana et al., 1994; Jimenez et al., 2002).

**Figure 2:**
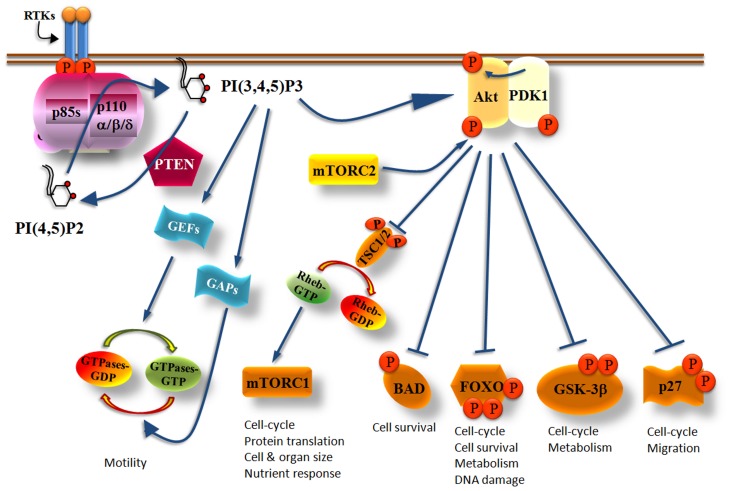
**Simplified scheme showing the critical role of Class IA PI3Ks in multiple cellular functions.** Upon PI3Ks activation, PI(3,4,5)P3 is produced and recruits Akt at the cell membrane where it becomes phosphorylated at The308 by PDK1 and at Ser473 by mTORC2. Fully activated Akt phosphorylates a variety of effector molecules including the TSC1/2, BAD, FOXO, GSK-3β, and p27 which then control cell growth, metabolism, survival, cell cycle, or migration. PI(3,4,5)P3 also activates GEFs or GAPs which then regulate the activity of small GTPases controlling cell motility. The PI(3,4,5)P3 levels produced by PI3Ks are regulated by the PTEN phosphatase which counteracts the PI3K reaction by dephosphorylating the 3-position of the inositol ring of PI(3,4,5)P_3_ yielding back PI(4,5)P_2_.

Activation of tyrosine kinase receptors by growth factors recruits class IA PI3 kinases to the cell membrane. Activated growth factor receptors possess phosphorylated Tyr-X-X-Met motifs in which bind with high affinity the regulatory subunits of class IA PI3Ks by their SH2 domains (Figure 1). In cells, activated class IA PI3Ks phosphorylate primarily the phosphatidylinositol (PI)-4,5-bisphosphate [PI(4,5)P2] yielding the product PI(3,4,5)P_3_ (Vanhaesebroeck et al., 2001) (Figure 2). The generation of PI(3,4,5)P_3_ leads to the recruitment of adaptor and effector proteins containing pleckstrin-homology (PH)-domains, including regulators of small GTPases [such as guanosine nucleotide exchange factors (GEFs) and GTPase-activating proteins (GAPs)] and Ser/Thr kinases (such as PDK1 and Akt/PKB), which thus become located at the plasma membrane (Klarlund et al., 1997; Krugmann et al., 2002; Welch et al., 2002; Marone et al., 2008). Small GTPases are activated (become GTP-bound) by GEFs whereas the return from their active state to an inactive state (GDP-bound) is catalyzed by GAPs (Figure 2). Cyclic activation-inactivation of the small GTPases is required for cell body to move properly (Ridley et al., 2003). PDK1, which is in an active state under basal conditions, becomes additionally activated on cell stimulation (Alessi et al., 1997a; Pullen et al., 1998; Currie et al., 1999) and phosphorylates Akt on Thr308 (Alessi et al., 1997a,b; Stokoe et al., 1997; Stephens et al., 1998). Akt is also phosphorylated on Ser473 (Alessi et al., 1996) by mTORC2 (mTOR complexed with the Rictor protein) (Sarbassov et al., 2005) (Figure 2). Full activation of Akt kinase activity requires the phosphorylation of both kinase domains of Akt (Bellacosa et al., 1998).

Akt activates or inhibits a broad range of proteins including mTORC1 (mTOR in complex with Raptor), BAD, FOXO, GSK-3β, and p27, which are involved in the control of cell growth, metabolism, survival, cell cycle, and migration (Manning and Cantley, 2007) (Figure 2). Akt phosphorylates and inactivates the tuberous sclerosis complex 1/2 (TSC1/2) which acts as a GAP protein on Ras homologue enriched in brain (RHEB), a guanosine triphosphate (GTP)-binding protein (Garami et al., 2003; Li et al., 2004). The role of GTP-bound RHEB is to activate mTORC1 and consequently its downstream effector proteins (Inoki et al., 2003). Thus, increased Akt activity promotes the activation of mTORC1 because Akt inactivates TSC1/2 (Figure 2). The multiple roles of mTORC1 and especially those correlated with mRNA translation and cell cycle has made the PI3K/Akt/mTORC1 axis an attractive target for the development of dual PI3K/mTOR inhibitors, mTOR-selective inhibitors and Akt inhibitors as anti-cancer drugs (Marinov et al., 2007; Sabbah et al., 2011; Castillo et al., 2012; Sheppard et al., 2012; Weigelt and Downward, 2012; Willems et al., 2012).

Akt also phosphorylates the death promoter BAD leading to the release of the anti-apoptotic proteins Bcl-2 and Bcl-XL (Datta et al., 1997; Peso et al., 1997). GSK-3 which regulates glucose metabolism and apoptosis is also controlled by Akt (Jope and Johnson, 2004). Phosphorylation of GSK-3β by Akt prevents its activity leading to the accumulation of cyclin D1 and the consequent transition of cells from G1 to the S phase of the cell cycle (Liang and Slingerland, 2003). Other substrates of Akt are the class O of transcription factors (FOXOs) that are known regulators of the cell cycle. Phosphorylated FOXOs bind to the 14-3-3 proteins resulting in the exclusion of FOXOs of the nucleus which leads to the increased transcription of cyclin D1 and to the reduced transcription of the p27 CDK inhibitor (CKI) (Alvarez et al., 2001; Burgering and Medema, 2003). Upon cytosolic localization of FOXOs also the transcription of FasL is prevented leading to the blockage of apoptosis. Akt also regulates post-translationally the p21 and p27 CKIs by phosphorylating them resulting in their exclusion of the nucleus (Zhou et al., 2001; Fujita et al., 2002; Liang et al., 2002) which consequently leads to increased cell proliferation due to decreased inhibition of cyclins. p27 acts as an oncoprotein in the cytoplasm where it binds to and inhibits RhoA thus promoting cell migration (Besson et al., 2004). The cytoplasmic localization of p21 and p27 is associated with high tumor grade, tumor cell invasiveness and metastasis (Sáez et al., 1999; Slingerland, 2000; Philipp-Staheli et al., 2001).

The PI3K/Akt signaling pathway is regulated by phosphatases with the phosphatase and tensin homologue deleted on chromosome 10 (PTEN) lipid phosphatase being the most extensively investigated. The PTEN tumor suppressor protein antagonizes the PI3K activity by dephosphorylating the 3-position of the inositol ring of PI(3,4,5)P_3_ (Maehama and Dixon, 1998) (Figure 2) thus controlling cell survival (Stambolic et al., 1998; Leslie and Downes, 2002; Sulis and Parsons, 2003). Reduced or lost activity of PTEN creates a state in which PI(3,4,5)P_3_ production is misregulated contributing to the constitutive activation of the PI3K pathway (Leslie and Downes, 2004; Parsons, 2004; Sansal and Sellers, 2004; Cully et al., 2006) and to abnormal cell growth (Ali et al., 1999; Vivanco and Sawyers, 2002; Luo et al., 2003).

The tissue distribution and the regulation of class IA PI3Ks expression have been determined using various approaches (Kok et al., 2009a,b). Reporter mice with a β-Gal-LacZ reporter gene inserted into endogenous p110 loci by homologous recombination were proven very useful in determining the distribution of p110α (Foukas et al., 2006) and p110δ (Okkenhaug et al., 2002; Eickholt et al., 2007). Whereas p110α and p110β were found to be globally expressed (Hu et al., 1993; Bi et al., 1999, 2002; Geering et al., 2007), p110δ is predominantly expressed in white blood cells (Chantry et al., 1997; Vanhaesebroeck et al., 1997b). p110δ is also expressed at high levels in some cancer cell lines and human tissues of non-leukocyte origin such as breast cancer cells (Sawyer et al., 2003; Tzenaki et al., 2012) and at moderate levels in neurons (Eickholt et al., 2007). The mechanism by which the expression of p110δ PI3K is regulated has recently been explored (Edwards et al., 2009; Kok et al., 2009b; Calvanese et al., 2012; Whitehead et al., 2012). A highly conserved transcription factor binding cluster in the *PI3KD* gene was identified and found to display higher promoter activity in leukocyte compared to non-leukocyte cells providing an explanation for the highly enriched p110δ levels in leukocytes (Kok et al., 2009b; Whitehead et al., 2012). Transcriptional regulation of *PIK3CD* by RUNX1 (Edwards et al., 2009) and leukocyte-dependent promoter DNA hypomethylation (Calvanese et al., 2012) were also proposed to be involved in high p110δ expression. It is possible that p110δ expression is transcriptionally regulated also in non-leukocyte cells that express high levels of p110δ, such as breast cancer cells, by leukocyte-related transcription factors which have been found to be activated in breast cancers (Teschendorff et al., 2007).

The three isoforms of class IA PI3K have identical enzymatic activities but they have non-redundant functions in cell signaling, metabolism, and tumorigenesis (Roche et al., 1994, 1998; Vanhaesebroeck and Waterfield, 1999; Hill et al., 2000; Hooshmand-Rad et al., 2000; Leverrier et al., 2003; Vanhaesebroeck et al., 2005; Foukas et al., 2006; Ali et al., 2008; Graupera et al., 2008; Papakonstanti et al., 2008). Since cancer-specific gain-of-function mutations were reported in *PIK3CA* gene (Campbell et al., 2004; Samuels and Velculescu, 2004), which encodes the p110α PI3K, this isoform has been placed in the center of cancer research. In contrast, no somatic mutations of genes encoding p110β or p110δ have been reported (Samuels and Velculescu, 2004; Thomas et al., 2007; Wood et al., 2007; Parsons et al., 2008; TGCA, 2008). Gene targeting and pharmacological studies have revealed a key role of p110β in platelet biology and thrombosis (Jackson et al., 2005) whereas recent studies have also shown a role of p110β in certain cancers and especially in tumor cells lacking PTEN (Ciraolo et al., 2008; Jia et al., 2008; Torbett et al., 2008; Wee et al., 2008; Zhu et al., 2008). Given that p110δ is preferentially expressed in leukocytes, the functional role of p110δ has been studied in immune system (Clayton et al., 2002; Jou et al., 2002; Okkenhaug et al., 2002; Ali et al., 2004; Aksoy et al., 2012) and this isoform has been more considered as target in immunity and inflammation (Rommel et al., 2007; Rommel, 2010; Soond et al., 2010). However, findings have been accumulated showing a seminal role of p110δ PI3K in lymphoid and myeloid malignancies. Furthermore, p110δ-selective inhibitors have entered clinical studies showing effective clinical outcomes in some hematologic malignancies (Fruman and Rommel, 2011; Castillo et al., 2012). Further data have also suggested a promising role of p110δ PI3K in oncogenesis and cancers of non-hematopoietic origin (Knobbe and Reifenberger, 2003; Mizoguchi et al., 2004; Boller et al., 2008; Zhao and Vogt, 2008a; Jia et al., 2009; Vogt et al., 2009; Jiang et al., 2010; Tzenaki et al., 2012). The malignancies with aberrant p110δ signaling that will be discussed below are summarized in Table 1.

**Table 1:** **Malignancies with aberrant p110δ signaling highlighted in this review**.

	**Malignancy**	**p110δPI3K aberration**
Hematological malignancies	Chronic lymphocytic leukemia (CLL)	Over-activation of p110δ signaling
	Multiple myeloma (MM)	
	Diffuse large B-cell lymphoma (DLBCL)	
	Hodgkin's lymphoma (HL)	
	Acute myeloid leukemia (AML)	
	Acute promyelocytic leukemia (APL)	
Solid non-hematologic tumors	Glioblastoma	Overexpression of p110δ mRNA, increased copy number of *PIK3CD*
	Prostate carcinoma	Overexpression of p110δ mRNA
	Neuroblastoma	Overexpression of p110δ protein
	Breast carcinoma	

In this review, we go over the main points of the evidence showing the critical role of p110δ PI3K in hematopoietic cells and malignancies, we highlight findings suggesting an emerging role of p110δ in non-hematologic cancers and discuss how a better understanding of p110δ regulation and function might reveal cancer contexts in which p110δ-selective inhibitors alone or in combination with inhibitors of other components of PI3K pathway could be beneficial.

## Role of p110δ PI3K in B cells and B-cell malignancies

B cells express all isoforms of the class I PI3K catalytic subunit (Bilancio et al., 2006), however, the p110δ PI3K was found to play a predominant role in most of the functions of B cells. The role of p110δ in B-cell development has been demonstrated by studies in p110δ knock-out (KO) and p110δ knock-in (KI) mice (Clayton et al., 2002; Jou et al., 2002; Okkenhaug et al., 2002; Beer-Hammer et al., 2010; Ramadani et al., 2010). These mice comprise significantly reduced numbers of mature circulating B cells because of delayed B cell maturation at the pro-B cell stage within the bone marrow whereas in B cells that eventually become mature the chemokine-induced migration, B-cell receptor (BCR) signaling, and BCR-induced proliferation were found to be impaired (Clayton et al., 2002; Jou et al., 2002; Okkenhaug et al., 2002; Reif et al., 2004). Although, the class IB p110γ PI3K is not essential for B-cell development (Sasaki et al., 2000), combined inactivation of both p110γ and p110δ led to greater reduction of peripheral B-cell numbers than p110δ inactivation alone, suggesting that p110γ and p110δ may have overlapping functions in B-cell development (Beer-Hammer et al., 2010). Studies using mice with p110α deficiency in lymphocytes showed that p110α is not essential for B cell development and BCR signaling, however, deletion of both p110α and p110δ isoforms blocked B cell development suggesting that only p110δ is required for antigen-dependent B-cell activation triggered by the BCR whereas p110α contributes to antigen independent tonic pre-BCR and BCR signaling (Ramadani et al., 2010). Lymphocyte-specific inactivation of p110β or combined inactivation of p110β and p110δ did not affect B cell development and activation (Ramadani et al., 2010).

**Figure 3:**
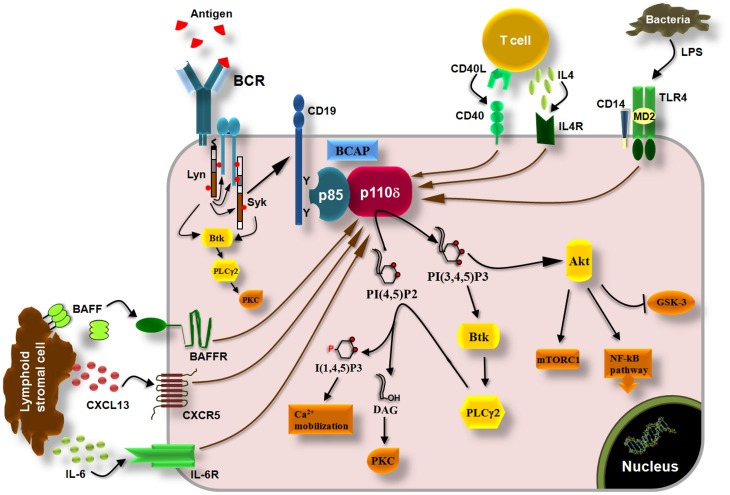
**p110δ PI3K pathway in B cells.** Engagement of BCRs by antigen induces the phosphorylation of ITAMs in the cytoplasmic tails of Ig-α and Ig-β by Lyn leading to recruitment and activation of Syk via ITAMs and to initiation of downstream signaling cascade. Tyrosine phosphorylation of the co-receptor CD19 and BCAP recruits p110δ PI3K through SH2 interactions leading to production of PI(3,4,5)P3 which recruits PH-domain containing proteins such as Akt, Btk, and PLCγ2. Akt controls the activity of multiple signaling molecules and pathways such as the mTORC1, GSK-3, and the NF-kB pathway. Btk phosphorylates and activates PLCγ2 which then catalyzes the hydrolysis of PI(4,5)P2 yielding I(3,4,5)P3 and diacylglycerol (DAG). I(3,4,5)P3 initiates Ca2+ mobilization and DAG induces the activation of protein kinase C (PKC) isoforms. The p110δ PI3K also functions downstream of the cytokine receptors BAFFR and IL6R, which are activated by BAFF and IL-6, respectively, derived from lymphoid stromal cells, and downstream of the IL4R which is activated by IL-4 derived from T cells. Chemokine receptors (CXCR5) and co-stimulatory receptors (CD40, TLRs) also induce the activation of p110δ PI3K in B cells.

In resting B cells, the TC21 GTPase was found to recruit the p85α/p110δ PI3K to non-phosphorylated BCR immunoreceptor tyrosine-based activation motifs (ITAMs) (Delgado et al., 2009). Although this finding is not supported by the data suggesting a role of p110α in antigen-independent pre-BCR and BCR survival signals (Ramadani et al., 2010), it is possible that both p110α and p110δ are recruited to the BCR by TC21, however, this remains to be determined. Upon antigen binding to BCR, the ITAMs in the cytoplasmic tails of Ig-α and Ig-β (Reth, 1992) are tyrosine phosphorylated by Lyn leading to recruitment and activation of Syk initiating thus the downstream signaling cascade (Kurosaki et al., 1994; Beitz et al., 1999) (Figure 3). The tyrosine phosphorylation of the scaffolding proteins CD19 and B-cell adaptor protein (BCAP) creates Src-homology 2 (SH2)-binding domains which allow the binding of the SH2 domains of the p85 subunit and the recruitment of the p85/p110 complex to the cell membrane (Tuveson et al., 1993; Fujimoto et al., 2000; Okada et al., 2000; Yamazaki et al., 2002; Aiba et al., 2008) (Figure 3). Downstream of PI3K, the BCR signaling pathways include the activation of Akt which then regulates the GSK-3, mTOR, and NF-kB pathway as well as the activation of Bruton's tyrosine kinase (Btk), which then induces the activation of phospholypase C-γ (Spaargaren et al., 2003; Fruman, 2004) (Figure 3). PLCγ is an enzyme that catalyzes the hydrolysis of PI(4,5)P2 to generate the second messengers inositol 1,4,5-trisphosphate [I(1,4,5)P3] and diacylglycerol (DAG) that regulate the reorganization of cytoskeleton and cell adhesion by inducing an increase in intracellular free-calcium levels and the activation of multiple protein kinase C (PKC) isoforms (Figure 3) and downstream signaling molecules (Papakonstanti et al., 2000; Fruman, 2004). The p110δ PI3K signaling pathway is also activated by cytokines like BAFF and IL6 (Patke et al., 2006; Henley et al., 2008) and chemokines like CXCL13 (Reif et al., 2004) derived from lymphoid stromal cells, by cytokines like IL-4 derived from T cells (Bilancio et al., 2006) and by co-stimulatory receptors such as CD40 and Toll-like receptors (TLRs) (Arron et al., 2001; Ni et al., 2012; So and Fruman, 2012; Troutman et al., 2012) (Figure 3). The mechanism that links the activation of p110δ to G protein-coupled chemokine receptors (such as the CXCR5) is not currently known. That p110δ PI3K mediates the effects of multiple receptors on B cells is consistent with substantial evidence documenting a central role of this enzyme in B cell development and activation (Okkenhaug and Fruman, 2010).

The critical role of p110δ in homeostasis and function of B cells combined with the fact that *PIC3CA* and *PTEN* gene mutations are rare in B cell malignancies (Leupin et al., 2003; Georgakis et al., 2006; Ismail et al., 2010) made the p110δ PI3K pathway to attract the interest in understanding its potential involvement in malignant B cells. Indeed, there is mounting evidence showing that the p110δ PI3K pathway is over-activated in B cell malignancies because of alterations in BCR signaling and other signals provided by factors from tumor microenvironment (Pauls et al., 2012). Significantly higher levels of p110δ PI3K activity have been determined in cells from patients with chronic lymphocytic leukemia (CLL) compared with normal hematopoietic cells (Herman et al., 2010) whereas overactivation of p110δ has also been found in multiple myeloma (MM) cell lines and cells from patients with MM (Ikeda et al., 2010) as well as in cell lines and cells from patients with Hodgkin's lymphoma (HL) (Meadows et al., 2012).

The critical role of p110δ in B cells led to the development of highly p110δ-specific inhibitors for treatment of B-cell malignancies (Norman, 2011) including the initially developed CAL-101 (GS-1101) (Fruman and Rommel, 2011; Lannutti et al., 2011). The activity of CAL-101 and other p110δ-selective inhibitors have been studied in cell lines and patient cells from different B-cell malignancies including CLL, MM, diffuse large B-cell lymphoma (DLBCL), and HL (Herman et al., 2010; Ikeda et al., 2010; Hoellenriegel et al., 2011; Lannutti et al., 2011; Meadows et al., 2012) (Table 2). Inhibition of p110δ PI3K by CAL-101 in cells from patients with CLL led to inhibition of Akt and ERK and consequently to reduced B-CLL survival (Herman et al., 2010; Hoellenriegel et al., 2011). Furthermore, inhibition of p110δ leads to blockade of protective microenvironmental signals on B-CLL. Indeed, survival signals induced by *in vitro* stimulation of B-CLL with TNFα, BAFF and CD40L were attenuated by CAL-101 treatment (Herman et al., 2010). The protective effect provided in B-CLL by their culture on fibronectin or stomal cells or their co-culture with nurse-like cells (NLC) was also blocked by inhibition of p110δ activity (Herman et al., 2010; Hoellenriegel et al., 2011). In these co-culture systems, elimination of p110δ activity additionally led to decreased secretion of the chemokines CCL2, CCL3 from CLL cells, CXCL13 from stromal cells, various survival factors from NLC and inhibited the chemotaxis of B-CLL to CXCL12, CXCL13 and stromal cell lines (Hoellenriegel et al., 2011). The later result is in line with previous findings showing that B cells derived from p110δ KO mice poorly respond to CXCL13 and exhibit reduced homing to lymphatic tissues (Reif et al., 2004). Similarly, the p110δ selective inhibitor IC87114 (Sadhu et al., 2003) inhibited the B cell chemotaxis to CXCL13 and S1P *in vitro* and led to depletion of MZ B cells from the spleen *in vivo* (Durand et al., 2009). A potential involvement of p110α, p110β, and p110γ in CLL has been indicated by a study showing that pharmacological inhibition of each of these isoforms inhibited the proliferation of CLL cells (Shehata et al., 2010).

**Table 2:** **Summarized findings showing the effects of p110 isoform-selective inhibitors in cell lines and patient cells from different hematological malignancies and in animal models described in the text**.

**Malignancy**	**Targeted isoform**			
	**p110δ**	**p110α**	**p110β**	**p110γ**
B-CLL	Inhibition of p-Akt and p-ERK Inhibition of B-CLL survival Inhibition of TNFα-, BAFF- and CD40L-induced survival Inhibition of protective effects provided by fibronectin, stomal cells or NLC Decreased secretion of CCL2 and CCL3 from CLL cells, CXCL13 from stromal cells, various survival factors from NLC Inhibition of chemotaxis of B-CLL to CXCL12, CXCL13, S1P and stromal cell lines Depletion of MZ B cells from the spleen	• Decreased proliferation of CLL cells	• Decreased proliferation of CLL cells	• Decreased proliferation of CLL cells
References	Durand et al. (2009), Herman et al. (2010), Hoellenriegel et al. (2011)	Shehata et al. (2010)	Shehata et al. (2010)	Shehata et al. (2010)
MM	Inhibition of phosphorylation of Akt and P70S6K Inhibition of MM cells growth Inhibition of IL6-, IGF-1- or stromal cells-induced phosphorylation of Akt, P70S6K and cell growth Inhibition of growth of transplanted human MM cells in xenograft mouse models	Not determined	Not determined	Not determined
Reference	Ikeda et al. (2010)			
DLBCL	Inhibition of phosphorylation of Akt and S6 Increased cleavage of the apoptotic markers caspase 3 and poly (ADP-ribose) polymerase	Not determined	Not determined	Not determined
Reference	Lannutti et al. (2011)			
HL	Inhibition of phosphorylation of Akt Induction in apoptosis Inhibition of stroma cells-induced Akt activation Disruption of survival signals mediated by CCL5, CCL17, and CCL22 in co-cultures of HL cells with stromal cells	Not determined	Not determined	Not determined
Reference	Meadows et al. (2012)			
AML	Inhibition of Akt phosphorylation Reduction in viable cells number Reduction in NF-kB activity Enhancement of cytotoxic effects of VP16	• Reduction in AML blast colony forming cells	• Modest effect in AML blast colony forming cells	• Modest effect in AML blast colony forming cells
References	Sujobert et al. (2005), Billottet et al. (2006)	Xing et al. (2012)	Xing et al. (2012)	Xing et al. (2012)
APL	Suppression of the ATRA-induced phosphorylation of Akt and S6 Induction in apoptosis	• No effect	Suppression of the ATRA-induced phosphorylation of Akt and S6 Induction in apoptosis	• No effect
References	Billottet et al. (2009)	Billottet et al. (2009)	Billottet et al. (2009)	Billottet et al. (2009)

The above summarized data suggested that inhibition of p110δ by CAL-101 reduces the B-CLL survival driven by B-cell molecules and furthermore acts by blocking cells to access protective niches inhibiting the environmental protective interactions that otherwise would promote B-cell survival and proliferation. These were promising results for potential efficacy of p110δ inhibition in CLL patients since CLL is characterized by the accumulation of B lymphocytes in the peripheral blood, lymph nodes, and bone marrow (Cheson et al., 1996; Hallek et al., 2008). Indeed, the phosphorylation of Akt in B-CLL and the plasma levels of CXCL13, CCL3, CCL4, and TNFα were found to be significantly reduced in patients treated with CAL-101 (Hoellenriegel et al., 2011; Sharman et al., 2011) suggesting that inhibition of p110δ disrupts the interactions of B-CLL from their protective microenvironment. These results are consistent with the increased numbers of B-CLL found in the peripheral blood of the CAL-101-treated patients (Sharman et al., 2011) indicating a release of lymphocytes from lymphoid tissues or a failure to home into lymph nodes which consequently leads to reduced lymph node size (Hoellenriegel et al., 2011; Castillo et al., 2012). It seems therefore that inhibition of p110δ in B-CLL *in vivo* is more efficient to release B-CLL from their microenvironment than to kill them which is consistent with recent data showing that even extremely reduced levels of class I PI3K activity are sufficient to sustain cell survival (Foukas et al., 2010). Inhibitors of Syk or Btk that also involved in BCR signaling (Figure 3) led to similar clinical responses (Herman et al., 2011; Ma and Rosen, 2011; Burger, 2012; De Rooij et al., 2012; Ponader et al., 2012; Puri and Gold, 2012).

Similar effects of CAL-101 and other p110δ inhibitors have been observed on other B-cell malignancies such as MM, DLBCL, and HL (Ikeda et al., 2010; Lannutti et al., 2011; Meadows et al., 2012) (Table 2). The important role of p110δ in MM pathogenesis was indicated by its high expression in patient MM cells (Ikeda et al., 2010). Suppression of p110δ expression or inhibition of p110δ activity by CAL-101 in MM cell lines and patient MM cells decreased the phosphorylation of Akt and P70S6K and inhibited cells growth (Ikeda et al., 2010). The implication of p110δ in protective signals derived from bone marrow microenvironment seems to be the case also in MM since CAL-101 inhibited MM cells growth and Akt and P70S6K phosphorylation in cells treated with IL-6 and insulin growth factor-1 (IGF-1) or co-cultured with bone marrow stromal cells (Ikeda et al., 2010). The effects of p110δ inhibition was also confirmed in two xenograft mouse models of human MM, where p110δ-inhibors prevented the growth of transplanted human MM cells and prolonged the host survival (Ikeda et al., 2010). Inhibition of p110δ activity by CAL-101 in DLBCL cell lines reduced the phosphorylation of Akt and S6 and increased the cleavage of the apoptotic markers caspase 3 and poly(ADP-ribose) polymerase (Lannutti et al., 2011). CAL-101 also decreased the phosphorylation of Akt and induced apoptosis in HL cell lines and moreover blocked the stroma cells-induced Akt activation in HL cells and disrupted the survival signals mediated by CCL5, CCL17, and CCL22 in co-cultures of HL cells with stromal cells (Meadows et al., 2012).

Various mechanisms that alter the activity of protein tyrosine kinases and phosphoinositide phosphatases that are involved in BCR signaling have been proposed to account for the over-activation of the PI3K pathway in malignant B-cells. Lyn kinase was found to be over-expressed in CLL and its inhibition led to induced apoptosis (Contri et al., 2005; Trentin et al., 2008). Syk is also over-expressed and constitutively phosphorylated and activated in CLL (Buchner et al., 2009; Efremov and Laurenti, 2011). The zeta-associated protein of 70-kD (ZAP-70) kinase, which is a Syk family kinase, is expressed in a subset of B-CLL patients (Rosenwald et al., 2001) and has been implicated in the elevated PI3K activity since its introduction in B cells that do not express ZAP-70 led to increased Akt phosphorylation (Gobessi et al., 2007). Other experiments have revealed that ZAP-70 functions as an adaptor protein in BCR signaling (Chen et al., 2008) and that the phosphorylation of Syk, PLCγ, and BLNK is enhanced in B-cell ZAP-70 positive compared to B-cell ZAP-70 negative CLL (Chen et al., 2005) which could indirectly alter the PI3K activity. B cells from ZAP-70 positive CLL patients were also found to express decreased levels of the SHIP phosphatase which affects PI3K signaling by dephosphorylating the product of PI3Ks PI(3,4,5)P3 producing PI(3,4)P2 (Brauweiler et al., 2000). The lipid phosphatase PTEN which directly antagonizes the PI3K pathway (Maehama and Dixon, 1998) has been found to be rarely mutated in B cell malignancies (Grønbæk et al., 1998; Sakai et al., 1998), however, its expression was found to be reduced or lost in CLL (Leupin et al., 2003). This has been attributed to be a result of miR-17-92 overexpression which negatively regulates PTEN expression in various leukemias (Lenz et al., 2008; Rao et al., 2012). Reduced PTEN activity has also been found in CLL (Shehata et al., 2010) which might be a result of overexpression and overactivation of CK2 that were also detected in CLL and blockade of CK2 decreased PTEN phosphorylation leading to PTEN activation (Shehata et al., 2010; Martins et al., 2011). Other than the control of PTEN activity by CK2 (Torres and Pulido, 2001), a variety of mechanisms regulate the activity of the PTEN tumor suppressor in B cells (Pauls et al., 2012) and might also be involved in B-cell malignancies, a possibility that remains to be determined.

## Role of p110δ PI3K in myeloid malignancies

Besides the B-cell malignancies, the role of class IA PI3Ks has been also studied in some myeloid malignancies (Table 2). Acute myeloid leukemia (AML) is characterized by the uncontrolled survival and proliferation of immature myeloid cells and their abnormal accumulation in the bone marrow. PI3K/Akt pathway was found to be constitutively activated in leukemic cells of AML patients, contributing to unrestricted cell survival and proliferation (Min et al., 2003; Xu et al., 2003; Zhao et al., 2003; Doepfner et al., 2007). Further studies have demonstrated that p110δ was the main PI3K isoform that was involved, as it was indicated from the higher p110δ expression levels, compared to other isoforms, in blast cells of AML patients (Sujobert et al., 2005). Treatment of these cells with the p110δ-specific inhibitor IC87114, suppressed the constitutive Akt activation (Sujobert et al., 2005; Billottet et al., 2006) to equal levels as those observed upon the pan-PI3K inhibitor LY294002 treatment (Sujobert et al., 2005) confirming that the p110δ is the main isoform contributor of PI3K activity in AML cells. It is of note that IC87114 did not affect the proliferation of normal hematopoietic progenitor cells (Sujobert et al., 2005). The combination of IC87114 with other antineoplastic agents such a topoisomerase II inhibitor VP16, which is used in AML patients treatment, further reduced AML cell numbers and NF-kB activity and most profoundly induced apoptosis (Billottet et al., 2006). Thus, the use of p110δ-specific inhibitors in combination with other cytotoxic drugs, may offer the maximum therapeutic efficiency in AML pathology accompanied with minimum overall toxicity. A recent study has shown that inhibition of p110α is also effective in killing AML blast colony forming cells (Xing et al., 2012), however, the concentration of p110α inhibitor used in this study was much higher (more than 1000 fold higher) than the IC50 value of this compound (Hayakawa et al., 2007) making thus unclear whether this inhibitor retains its isoform selectivity. Inhibitors for p110β or p110γ had a very modest effect (Xing et al., 2012).

Acute promyelocytic leukemia (APL) is a relative to AML malignancy, characterized by the increased accumulation of abnormal promyelocytes to the bone marrow due to their inability to differentiate normally and to their increased resistance in apoptotic signals. Similar to AML, p110δ seems to contribute in the constitutive PI3K signaling observed in APL promyelocytes (Billottet et al., 2009). However, p110β is also involved in APL and in line with this, both isoforms are consistently expressed in APL cells (Billottet et al., 2009). All-trans retinoic acid (ATRA) is an agent used in APL treatment because of its potential to promote the differentiation of promyelocytic leukemic cells by regulating the PI3K/Akt/mTOR pathway (Lal et al., 2005). Inhibition of p110δ or p110β suppressed the ATRA-induced Akt and S6 phosphorylation without affecting the ATRA-induced differentiation (Billottet et al., 2009). Dual inhibition of both p110δ and p110β activity promoted the apoptosis of primary APL cells in the presence and in the absence of ATRA treatment (Billottet et al., 2009). These results suggested that inhibition of p110δ and p110β combined with differentiation induced treatments may represent potential therapeutic targets in APL.

The mechanism leading to the dominant activity of p110δ in myeloid malignancies is not yet completely clear. The most profound reason seems to be its high expression in leukocytes since no mutations on *PIK3CD* gene have been found in samples from AML patients (Cornillet-Lefebvre et al., 2005). The PTEN expression levels were also readily detected in AML samples and its phosphorylation on S380/T382/T383 was marginally affected (Billottet et al., 2006), indicating that other mechanisms are involved in the constitutive Akt activation in AML. The constitutive p110δ activity in AML could be triggered by upstream factors and autocrine mechanism. Previous studies had shown that insulin growth factor-1 receptor (IGF-1R) signaling is constitutively activated in AML cells through IGF-1 autocrine production (Doepfner et al., 2007; Tazzari et al., 2007). The IGF-1R was found to be constitutively phosphorylated in all leukemic cells tested, whereas its inhibition with neutralizing anti-IGF-1R strongly inhibited the phosphorylation of Akt and cell proliferation in AML cells (Chapuis et al., 2010). Inhibition of both p110β and p110δ impaired the IGF-1 stimulated Akt activation, cell growth and survival, suggesting that both isoforms are activated downstream of IGF-1 signaling in AML cells (Doepfner et al., 2007).

## A promising role of p110δ PI3K emerges in non-hematologic cancers

Since the p110δ PI3K was cloned and characterized (Chantry et al., 1997; Vanhaesebroeck et al., 1997b) an increasing catalog of evidence showing p110δ-isoform specific functions in hematopoietic cells have suggested p110δ as a potential therapeutic target in immunity, inflammation, and hematological malignancies (Rommel et al., 2007; Okkenhaug and Fruman, 2010; Rommel, 2010; Soond et al., 2010; Fruman and Rommel, 2011). The p110δ-isoform specific functions were demonstrated by mice with inactivated p110δ (Clayton et al., 2002; Jou et al., 2002; Okkenhaug et al., 2002; Ali et al., 2004; Aksoy et al., 2012) and by p110δ-selective inhibitors such as the IC87114 compound, which was the first isoform-selective inhibitor published (Sadhu et al., 2003), and the CAL101 which has recently entered clinical studies for hematologic malignancies (Fruman and Rommel, 2011; Castillo et al., 2012). It was the preferential expression of p110δ in leukocytes (Chantry et al., 1997; Vanhaesebroeck et al., 1997b) together with the absence of somatic mutations in *PIK3CD* gene that placed p110δ PI3K in the realm of immune system and hematologic cancers.

A non-expecting role of p110δ in oncogenesis of non-hematopoietic cells was first observed in avian fibroblasts in which overexpression of wild-type p110δ induced oncogenic transformation (Kang et al., 2006). The p110δ-overexpressing cells were found to express elevated levels of phosphorylated Akt, comparable to those detected in cells expressing the oncogenic H1047R p110α mutant (Kang et al., 2006). The p110δ oncogenic activity was not required binding of p110δ to RAS and was resistant to inhibitors of the MAPK pathway (Zhao and Vogt, 2008a; Vogt et al., 2009). Further data have also suggested a role of p110δ in non-hematologic human cancers (Table 1). Overexpression of p110δ mRNA and increased copy number of the *PIK3CD* gene were found in some cases of glioblastoma (Knobbe and Reifenberger, 2003; Mizoguchi et al., 2004). p110δ mRNA was also found to be increased in prostate carcinoma compared with normal prostate (Jiang et al., 2010). Abnormally high p110δ expression levels were found in primary neuroblastoma tissue compared with the normal adrenal gland tissue (Boller et al., 2008) and suppression of p110δ expression in neuroblastoma cells led to impaired cell growth and survival (Boller et al., 2008). All this evidence suggested that the expression levels of wild-type p110δ might correlate with its oncogenic potential.

**Figure 4:**
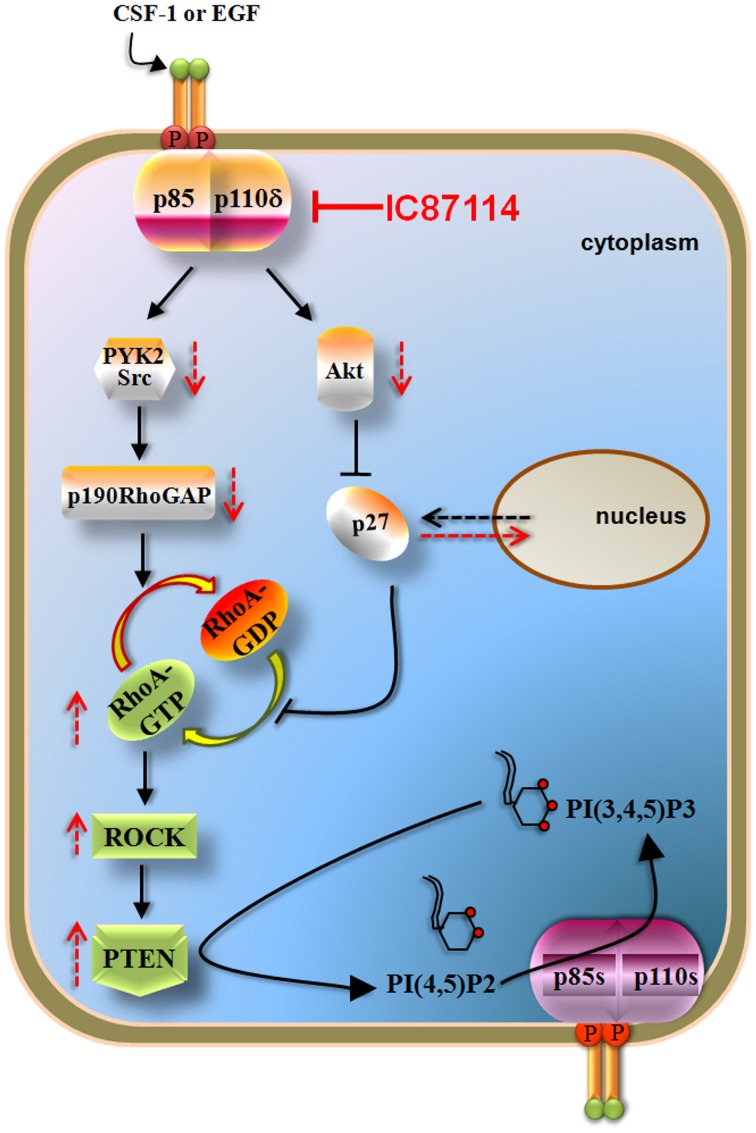
**p110δ PI3K mediates the effects of CSF-1 or EGF via RhoA and PTEN in macrophages and some solid tumor cells.** Activation of p110δ leads to increased activity of p190RhoGAP (through PYK2/Src activation) and to cytoplasmic accumulation of p27 (which is mediated by the increased Akt activity). p190RhoGAP induces the inactivation of RhoA whereas p27 prevents the activation of RhoA both thus leading to reduced RhoA activity and consequently to decreased PTEN activity. Inactivation of p110δ by IC87114 reverses these pathways leading to PTEN activation which then opposes the PI3K reaction of the remaining active p110 isoforms. The p190RhoGAP-driven mechanism almost solely keeps RhoA activity low under basal whereas that of p27 contributes upon stimulated conditions. Arrows with dashed lines represent alterations in activity or location in the presence (red) or in the absence (black) of IC87114.

Recent data documented that p110δ PI3K inhibits the activity of the PTEN tumor suppressor via a negative signaling pathway that involves inhibition of RhoA/ROCK (Papakonstanti et al., 2007) (Figure 4). The activation of p110δ PI3K was found to positively regulate the p190RhoGAP activity and to result in the accumulation of p27 in the cytoplasm (Papakonstanti et al., 2007). Given that p190RhoGAP catalyzes the return of RhoA-GTP (active state) to RhoA-GDP (inactive state) (Bernards and Settleman, 2005) and p27 prevents the return of RhoA-GDP to RhoA-GTP (Besson et al., 2004) the activation of p110δ leads to decreased RhoA activity and consequently to decreased PTEN activity. Upon genetic or pharmacological inactivation of p110δ by IC87114, PTEN becomes activated and dampens the PI3K pathway (Figure 4). The isoform-selective role of p110δ in the negative regulation of RhoA and the mechanism by which RhoA regulates PTEN activity under the control of p110δ are not currently understood but it seems that these are related with activation of p110δ at certain cellular compartments (Papakonstanti, unpublished data). This feedback mechanism was originally found to be the case in primary (Papakonstanti et al., 2007) and transformed macrophages (Papakonstanti et al., 2008) and in mouse brain tissue (Eickholt et al., 2007). More recently we showed that the negative regulation of PTEN by p110δ is also the case in those cancer contexts where p110δ is expressed at high levels (Tzenaki et al., 2012). The p110δ protein was found to be expressed at different levels in different cancer types e.g., the p110δ PI3K is the predominant isoform expressed in human primary breast carcinoma, whereas ovarian and cervical human carcinomas mainly express p110α and p110β (Tzenaki et al., 2012). The activity of wild-type PTEN was found to be suppressed in breast and prostate cancer cells that express high levels of p110δ suggesting that the elevated expression of p110δ might provide these cells with a competitive advantage to keep their wild-type PTEN inactive (Tzenaki et al., 2012). Breast and prostate cancer cells expressing functional PTEN were also sensitive to anti-proliferative effect of p110δ inhibitors through PTEN activation. In contrast, inhibition of p110δ in ovarian and cervical cancer cells which express very low levels of p110δ had no effect neither in PTEN activity nor in cell proliferation (Tzenaki et al., 2012).

The p110δ expression levels might therefore represent one of the parameters that correlate with the cancer type-specific response to PI3K pathway inhibitors, a possibility that will be important to be explored in future studies. This hypothesis is also corroborated by other published data. For example, breast cancer cells were found to be sensitive to growth inhibition by PI3K inhibitors without having mutations in *PTEN* or *PIK3CA* genes (O'Brien et al., 2010). On the other hand, breast cancer cells with PTEN deficiency were found to be resistant to PI3K inhibitors (Tanaka et al., 2011), PI3K/mTOR inhibitors (Brachmann et al., 2009) or mTOR inhibitors (Weigelt et al., 2011) whereas some of the PTEN-deficient breast cancer cell lines were sensitive to inhibitors of the PI3K pathway (She et al., 2008; Lehmann et al., 2011; Sanchez et al., 2011; Tanaka et al., 2011). In ovarian cancer cells, however, *PIK3CA* gain-of-function mutations and PTEN deficiency were correlated with their response to PI3K pathway inhibitors (Ihle et al., 2009; Di Nicolantonio et al., 2010; Meuillet et al., 2010; Santiskulvong et al., 2011; Tanaka et al., 2011; Meric-Bernstam et al., 2012). There are also evidence showing that in human breast tumor cells and cancer cell lines there is no good correlation between the presence of *PIK3CA* gain-of-function mutations and the basal or growth factor stimulated PI3K and Akt activity (Stemke-Hale et al., 2008) suggesting that other regulatory mechanism may affect the status of PI3K activity. It will be important to determine whether in breast cancers that *PIK3CA* gene is mutated and *PTEN* gene is wild-type, induction of PTEN activity by inhibition of p110δ PI3K dampens the production of PI(3,4,5)P_3_ and cell growth. An open question is also whether in cells with heterozygously mutated PTEN, the remaining wild-type PTEN allele is under the influence of high levels of p110δ. Given that *PTEN* gene is often wild-type in human breast cancers (Stemke-Hale et al., 2008; Chalhoub and Baker, 2009), further experiments may reveal that p110δ-selective inhibitors alone or combined with inhibitors of other components of PI3K pathway could be beneficial in this cancer type.

## Conclusions

The PI3K signaling pathway was brought at the center of attention in the field of cancer research by the discovery of cancer-specific gain-of-functions mutations in *PIK3CA* gene (Campbell et al., 2004; Samuels and Velculescu, 2004). Deregulated PI3K signaling in cancer has also been attributed to gain of function in receptor tyrosine kinases, activated Akt or to loss-of-function mutations in *PTEN* gene (Vivanco and Sawyers, 2002; Engelman et al., 2006; Yuan and Cantley, 2008). Although the p110α PI3K pathway and the loss of function of PTEN have received a great attention for their involvement in human cancers there are still some unexplained observations. Indeed, recent studies have shown that there is poor correlation between the *PIK3CA* or *PTEN* mutational status in cancer cell lines and the response of these cells to anti-proliferative effect of PI3K inhibitors (Edgar et al., 2010; O'Brien et al., 2010; Tanaka et al., 2011) indicating that unidentified mechanisms or PI3K isoform(s) other than p110α are also involved in the control of cancer cells survival. There are also evidence documenting no correlation between the oncogenic activity of p110α PI3K and signaling through Akt (Gymnopoulos et al., 2007; Zhao and Vogt, 2008b; Vasudevan et al., 2009) suggesting that Akt can be a non-obligatory partner in PI3K signaling and that Akt-independent PI3K pathways may be important in cancer cells.

The relationship of p110δ PI3K with cancer had received much less attention but recently p110δ has entered the realm of hematologic cancers and p110δ-selective inhibitors have provided promising results in some hematological malignancies (Fruman and Rommel, 2011; Castillo et al., 2012). p110δ is exceptional in that it regulates not only homeostasis and function of B-cells but also it is involved in the transduction of microenvironmental signals including chemokines and cytokines derived from lymphoid tissues or T-cells (Okkenhaug and Fruman, 2010; Puri and Gold, 2012). p110δ-selective inhibitors have been studied in multiple hematologic malignancies and the most promising results are currently available for B-CCL. It was remarkable that inhibition of p110δ in B-CLL cells or treatment of patients with the p110δ-selective inhibitor CAL-101 prevented B-CLL survival and moreover disrupted the signals from supporting cells of B-CLL microenvironment thus providing an anti-tumor activity (Herman et al., 2010; Hoellenriegel et al., 2011; Castillo et al., 2012). The fact, however, that CAL-101 inhibited cytokine production by human T cells (Herman et al., 2010; Hoellenriegel et al., 2011) together with data showing that p110δ plays an important role in functions of NK cells (Kim et al., 2007; Saudemont et al., 2007), in anti-tumor response of cytotoxic T lymphocytes (Putz et al., 2012) and in the development of regulatory T cells (Patton et al., 2006), raise the question if the efficacy of p110δ inhibition might be counterbalanced by a potential suppression of anti-tumor immunity. Nevertheless, although a single agent treatment was unexpected to have clinical activity, the outcome of the patients treated with CAL-101 went far beyond the expectations.

A critical role of p110δ in solid tumor cells has just emerged by published data showing that an oncogenic potential of p110δ might correlate with its expression levels (Knobbe and Reifenberger, 2003; Mizoguchi et al., 2004; Boller et al., 2008; Zhao and Vogt, 2008a; Vogt et al., 2009; Jiang et al., 2010) and by evidence documented that in those solid tumor cells expressing leukocyte-levels of p110δ, this isoform suppresses the activity of wild-type PTEN rendering these cells sensitive to growth-inhibitory effects of p110δ-selective inhibitors (Tzenaki et al., 2012). The mechanism that accounts for the high expression levels of p110δ PI3K in some cancer types whilst in others the expression of p110δ is very low (Tzenaki et al., 2012) is unclear at the moment. The differential expression of p110δ in human cancers might be a result of transcriptional regulation by differentially activated transcription factors in each cancer type or a consequence of epigenetic aberrations. It is also of note that the expression levels of the PI3K regulatory subunit p85β were found to be elevated in breast carcinomas and that altered *PIK3R2* expression affected tumor progression (Cortés et al., 2012). It will be important to determine whether a specific combination of p110δ with p85β exists in breast carcinomas and whether it affects the activity of p110δ.

There is also evidence to suggest that GEFs and GAPs, which regulate the activity of small GTPases, could play important roles in cancer biology as components of PI3K signaling. Indeed, P-REX2a (phosphatidylinositol 3,4,5-trisphosphate Rac exchanger 2a), which activates the small GTPase Rac, was found to interact with PTEN and directly inhibit PTEN function (Fine et al., 2009). It is also of note that the p190RhoGAP-driven mechanism (the p110δ/p190RhoGAP/RhoA/PTEN branch in Figure 4), contributes almost solely to inhibition of RhoA and PTEN under non-stimulated conditions (Papakonstanti et al., 2007) suggesting that p110δ can signal to a large extend independently of Akt through p190RhoGAP and RhoA.

The p110δ PI3K has just become eminent in the field of hematologic malignancies and it seems to have the potential to come into the spotlight of non-hematologic cancers. More work is needed to delineate the role of p110δ in cancer and to determine important aspects of its regulation and function such as the possible activation of p110δ at certain cellular locations, the mechanisms by which p110δ regulates its cellular locations, the role of p110δ in cancers cells expressing mutated p110α and/or heterozygously mutated PTEN, the p110δ triggered signaling through effectors other than Akt and last but not least the mechanism that regulates the differential expression of p110δ in different cancer types. Answering these questions will reveal a ground of discoveries that might illuminate some currently unexplained observations potentially improving therapeutic strategies against cancer.

### Conflict of interest statement

The authors declare that the research was conducted in the absence of any commercial or financial relationships that could be construed as a potential conflict of interest.
